# Catalytic-independent neuroprotection by SIRT1 is mediated through interaction with HDAC1

**DOI:** 10.1371/journal.pone.0215208

**Published:** 2019-04-11

**Authors:** Jason A. Pfister, Chi Ma, Santosh R. D’Mello

**Affiliations:** 1 Department of Biological Sciences, Southern Methodist University, Dallas, TX, United States of America; 2 National Institutes of Health, Bethesda, MD, United States of America; H Lee Moffitt Cancer Center and Research Institute, UNITED STATES

## Abstract

SIRT1, a NAD+-dependent deacetylase, protects neurons in a variety of *in vitro* and *in vivo* models of neurodegenerative disease. We have previously described a neuroprotective effect by SIRT1 independent of its catalytic activity. To confirm this conclusion we tested a panel of SIRT1 deletion mutant constructs, designated Δ1–Δ10, in cerebellar granule neurons induced to undergo apoptosis by low potassium treatment. We find that deletions of its N-terminal, those lacking portions of the catalytic domain, as well as one that lacks the ESA (*E*ssential for *S*IRT1 *A*ctivity) motif, are as protective as wild-type SIRT1. In contrast, deletion of the region spanning residues 542–609, construct Δ8, substantially reduced the neuroprotective activity of SIRT1. As observed with LK-induced apoptosis, all SIRT1 constructs except Δ8 protect neurons against mutant huntingtin toxicity. Although its own catalytic activity is not required, neuroprotection by SIRT1 is abolished by inhibitors of Class I HDACs as well as by knockdown of endogenous HDAC1. We find that SIRT1 interacts with HDAC1 and this interaction is greatly increased by deleting regions of SIRT1 necessary for its catalytic activity. However, SIRT1-mediated protection is not dependent on HDAC1 deacetylase activity. Although other studies have described that catalytic activity of SIRT1 mediates is neuroprotective effect, our study suggests that in cerebellar granule neurons its deacetylase activity is not important and that HDAC1 contributes to the neuroprotective effect of SIRT1.

## Introduction

Histone deacetylases (HDACs) are the catalytic subunits of multiprotein complexes that deacetylate specific lysines in the tail residues of histones, resulting in the compaction of chromatin into a transcriptionally repressed state (reviewed in [1, 2]). Although best studied for their effects on histones and transcriptional activity, it is now known that they regulate the acetylation status of a number of other non-histone proteins, suggesting complex functions for HDACs [1, 2]. Mammals express eighteen HDACs that make up two distinct groups, classical HDACs (HDAC1–11) that are Zn-dependent, and the sirtuins (SIRT1–7), which require NAD^+^ for activation. It is well established that classical HDACs regulate neurodegeneration with some members of the family promoting neurodegeneration whereas others protect against it [3, 4]. Similarly, sirtuins have also been described to have both protective and neurotoxic effects [5–8].

Among the sirtuins, SIRT1 is the best-studied in the context of neurodegeneration where it has been shown by multiple laboratories to have strong neuroprotective actions (reviewed in [6–8]). Indeed SIRT1 is highly protective in both *in vitro* and *in vivo* models of Alzheimer’s disease (AD), Huntington’s disease (HD), Parkinson’s disease (PD), amyotrophic lateral sclerosis (ALS), and the response to strokes [9–12]. Endogenous SIRT1 expression is decreased in Aβ cell culture and mouse models of AD [13]. However, stimulation of its expression or activation, such as by pharmacological means, can reduce Aβ deposits as well as improve cognitive deficits in these models [14–16]. This has been suggested to occur by SIRT1 controlling the proper cleavage of amyloid precursor protein (APP) and thus regulating the balance between amyloidgenic and non-amyloidgenic APP processing [7, 13–17]. SIRT1 protection against Aβ might also involve Aβ degradation by modulating autophagy [18]. Interestingly, SIRT1 is upregulated in mouse models of AD/tauopathies and ALS and provides a protective effect [10, 19]. In a mouse model of tauopathy, SIRT1 was shown to deacetylate tau, leading to tau degradation and a reduction in the spread of pathogenic tau [19, 20]. Similar to models of AD, in both *in vitro* and *in vivo* models of HD SIRT1 expression and activity can activate multiple targets and transcriptional pathways that regulate processes such as mitochondrial biogenesis, antioxidant defense, and neurotrophic support, thereby providing a protective effect against mutant Huntingtin (mut-Htt) [9, 21, 22]. However, mut-huntingtin and its aggregates can also interact with and inhibit SIRT1 deacetylase activity [9] leading to hyperacetylation of SIRT1 substrates. Thus, enhancing SIRT1 expression and its activity has clearly revealed it to be an attractive therapeutic approach for neurodegenerative disease. Understanding the mechanism by which SIRT1 protects could lead to the identification of additional therapeutic targets.

We previously described evidence suggesting that SIRT1 was able to protect neurons from death independent of its well-documented catalytic activity [5]. A recent study by Singh et al. also described that SIRT1 could protect SH-SY5Y neuroblastoma cells from rotenone toxicity and reduced α-synuclein aggregation through a catalytically-independent mechanism [11]. Furthermore, other functions of SIRT1 in non-neuronal cells can also be mediated independent of its catalytic activity [23–25]. These studies suggest that SIRT1 can function both through its enzymatic activity and through other mechanisms independent of it.

Here, we elaborate on our previous findings and show that protection by SIRT1 is mediated by a previously uncharacterized 67 amino acid region, termed here as Δ8, just C-terminal to SIRT1’s catalytic domain. While already shown to be protective against Huntington’s disease in mice, we show that increased SIRT1 expression is able to protect against mut-huntingtin toxicity in the same deacetylase-independent manner in cultured neurons. Protection by SIRT1 is not regulated by well-known pro-survival signaling pathways, but is blocked by classical HDAC inhibitors and knockdown of HDAC1.

## Materials and methods

### Materials

Unless specified otherwise, all tissue culture media was purchased from Invitrogen and all chemicals and reagents were from Sigma-Aldrich (St. Louis, MO). Poly-L-Lysine for primary neuronal cultures was from Trevigen (Gaithersburg, MD). Antibodies used in this study were: GFP (catalog # SC-9996, Sana Cruz Biotechnology, Dallas, TX and catalog # 50430-2-AP, Proteintech, Rosemont, IL), Flag (catalog # F1804 and # F7425, Sigma-Aldrich), IgG (catalog # sc-69786, Santa Cruz), SIRT1 (catalog # D1D7, Cell Signaling and catalog # 60303-1-lg, Proteintech), and HDAC1 (catalog # 66085-1-lg, Proteintech). Primary antibodies were used a concentration ranging from 1:1,000 to 1:20,000 in 5% bovine serum albumin. Fluorescent secondary antibodies for immunocytochemistry were from Jackson ImmunoResearch (West Grove, PA). HRP-conjugated secondary antibodies for western blot (from Piece Rockford, Rockford, IL) were used a 1:10,000 concentration. Enhanced polyvinylidene difluoride (PVDF) membrane was from Bio-Rad (Hercules, CA, USA).

### Expression plasmids

Expression plasmids used in this study were as follows: Flag-tagged full length SIRT1 and the ten deletion constructs (Δ1-Δ10) were a kind gift from Zhenken Lou at the Mayo Clinic. The following were purchased from Addgene: SIRT1 deacetylase-deficient mutant, H363Y, (#1792) was donated by Michael Greenburg, HDAC1-GFP (#11054) and HDAC1-Δ56-GFP (#11055) were donated by Ramesh Shivdasani, and HDAC3-Flag (#13819) was donated by Eric Verdin. Huntingtin constructs, Htt-GFP (Q15) and mut-Htt-GFP (Q138), contain the first exon of huntingtin (residues 1–588) with either 15 or 138 glutamine repeats, respectively, and were kind gifts from J. Troy Littleton at Massachusetts Institute of Technology. The pLK0.1-TRC (pLK0.1) control shRNA, which contains a non-hairpin 18 bp insert, was purchased from Addgene (#10879) and donated by David Root.

### Culture, transfection, and treatment of neurons

Cerebellar granule neurons (CGNs) were cultured as previous described [26] from 7–8 day old Wistar rats following rapid decapitation. Cytosine arabinoforanoside (10 μM) was added to the culture medium 18–22 h after plating to prevent replication of non-neuronal cells. Transfections were performed on day 5 *in vitro* by the calcium phosphate method as previously described [27, 28] and allowed to express for 24 h. Cultures were then switched to serum free culture medium (Basal Minimal Eagle’s medium, 2 mM glutamine and 0.2% gentamycin) supplemented with 25 mM KCl (High Potassium, HK) or without KCl (Low Potassium, LK). For pharmacological inhibitor studies, at the time of media switch cells were treated with either HK/LK medium or HK/LK medium supplemented with inhibitors of the following concentrations: PD98059 (MEK1, 50 μM), U0126 (MEK1/2, 10 μM), LY294002 (PI3-K, 10 μM), Wortmannin (PI3-K, 100 nM), IC261 (CK1, 10 μM), H89 (PKA, 3 μM), KN-62 (CaMKII, 10 μM), TSA (HDACs, 500 nM), pimelic diphenylamide 106 (Class I HDACs, 10 μM), RGFP966 (HDAC3, 10 μM), Roscovitine (CDKs, 50 μM), SP600125 (JNK, 10 μM), SB216763 (GSK3, 5 μM), and HSB-13 (25 μM) [29–34]. All pharmacological inhibitors were purchased from Calbiochem (Billerica, MA), except pimelic diphenylamide 106 and SP600125 that was from Sigma-Aldrich, and RGFP966 that was from Fisher scientific, and their ability to inhibit their targets at the doses listed above was confirmed in control experiments (data not shown). Following a 24 h treatment, cells were fixed and subjected to immunocytochemistry. Cell viability was quantified based on cell morphology using DAPI (4’6’-diamidino-2-phenylindole hydrochloride) staining as previously described [5]. Cells with condensed or fragmented nuclei were scored as dead. All work involving animals was approved by the IACUC at Southern Methodist University.

### Culture and transfection of cell lines

The HEK293T (catalog # CRL-11268) cell line was purchased from ATCC and maintained in DMEM supplemented with 10% FBS at 37°C and 5% CO_2_. All cell line transfections were performed using Endofectin (GeneCopoeia, Maryland) diluted in Opti-Mem reduced serum media by following the manufacturer’s guidelines.

### Immunoprecipitation and western blotting

For immunoprecipitation experiments, HEK293T cells were grown in 60 mm dishes to 85–90% confluency. Co-transfections were performed as just described in a 1:1 ratio for 48 h. For endogenous immunoprecipitation, CGNs were plated in 100 mm dishes and treated with HK/LK media for 6 h on day 6 *in vitro*. Cells were then lysed in 500 μl of 1x cell lysis buffer (20 mM Tris-HCl pH 7.5, 150 mM NaCl, 1 mM Na_2_EDTA, 1 mM EGTA, 1% Triton, 2.5 mM sodium pyrophosphate, 1 mM beta-glycerophosphate, 1 mM Na_3_VO_4_, 1 μg/ml leupeptin) containing a protease inhibitor cocktail tablet (Roche) in ice with periodic mixing for 20 min, and then centrifuged at 14,000 x g for 10 min at 4°C. The supernatant was collected as the whole-cell soluble lysate (WCL) and protein concentration was determined by the Bradford assay (Bio-Rad). 50 μg of WCL, referred to as the input, was mixed with 6x sodium dodecyl sulfate (SDS) sample buffer (375 mM Tris–HCl, pH 6.8, 12% SDS, 60% glycerol, 300 mM dithiothreitol, and 0.012% bromophenol blue) and then placed at -80°C. 500 μg of precleared WCL was rocked overnight at 4°C with 1 μg of pull-down antibody. 30 μl of Protein A/G Plus-Agarose beads (catalog # sc-2003, Santa Cruz) were then added to the WCL and rocked for 1–2 h at 4°C. Antibody-bound beads were then pelleted by centrifugation at 4,000 rpm for 2 min at 4°C, the supernatant was discarded, and the beads were washed three times with 500 μl of 1x cell lysis buffer. Following the final wash, pelleted beads where mixed with 30 μl of 3x SDS and, along with the input removed from -80°C, boiled at 95°C for 5 min and subjected to SDS–PAGE. Proteins were electrophoretically transferred from the gel to PVDF membrane at 4°C overnight. Membranes were incubated in blocking buffer (1x TBS, 5% w/v nonfat dry milk, and 0.05% Tween-20) at 25°C for 1 h, then subsequently incubated at 4°C overnight with primary antibodies, which was followed by secondary antibody for 1 h at 25°C. Immunoreactivity was developed by enhanced chemiluminescence and visualized with the Bio-Rad Chemi-Doc imaging system using Clarity western ECL substrate (Bio-Rad).

### shRNA-mediated knockdown

For knockdown experiments, the following shRNA targeting HDAC1 was purchased from Sigma-Aldrich and previously shown as effective in knocking down endogenous protein levels [35]: TRCN0000039400 referred to here as sh A. A pLK0.1 control shRNA or sh A was transfected into neuronal cultures, as described above, along with EGFP or SIRT1-Flag in a 2:1 ratio on day 4 *in vitro*. Medium was switched 48 h later to HK/LK media for 24 h. Viability was quantified by immunocytochemistry and DAPI staining based on EGFP or SIRT1 expression using GFP and Flag antibodies.

### Statistical analysis

All graphs were created and all statistical analysis was performed using GraphPad Prism software. Unless state otherwise, one-way ANOVA with Tukey’s multiple comparisons posttest was used to compare across multiple data sets. Results for each graph are shown as mean ± standard deviation from at least three independent experiments. *p* values of 0.05 were considered as significant and the following asterisk were used to denote statistical significance: * *p*<0.05, ^ *p*<0.05, ** *p*<0.01, ## *p*<0.01, *** *p*<0.001, ### *p*<0.001. For quantification of viability, ≥ 200 cells per coverslip were counted for each experiment.

## Results

### Neuroprotection by SIRT1 does not require its deacetylase activity

**Fig 1:**
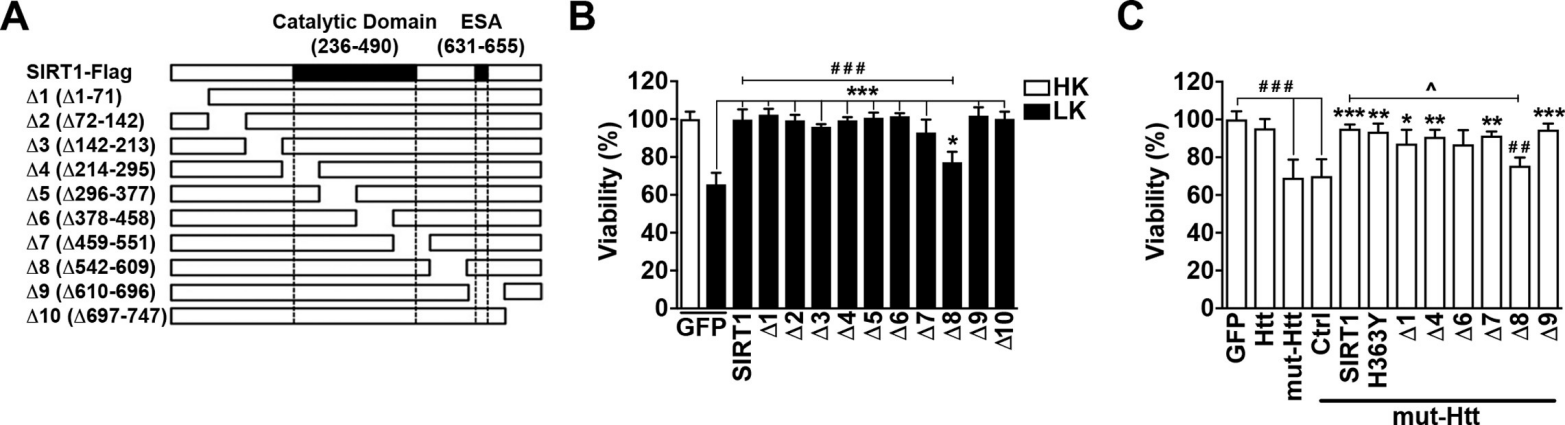
Neuroprotection by SIRT1 does not require its deacetylase activity. **A.** Schematic of the Flag-tagged SIRT1 and ten deletion constructs used in this study (adapted from [40]). ESA: *E*ssential for *S*IRT1 *A*ctivity motif. **B.** Full length SIRT1, the ten deletion constructs (Δ1-Δ10), and a GFP control were ectopically expressed in CGNs and treated with either HK or LK media. Viability was quantified as described in material and methods (* *p*<0.05 and *** *p*<0.001 as compared to GFP LK; ### *p*<0.001 compared to SIRT1; n = 3). **C.** Htt-GFP, mut-Htt-GFP, and a GFP control were ectopically expressed into CGNs. Additionally, mut-Htt was co-expressed in a 1:2 ratio with either a control vector (Ctrl, 3xFlag), full length SIRT1-Flag, a SIRT1 deacetylase mutant (H363Y-Flag), or Flag-tagged Δ1, Δ4, Δ6, Δ7, Δ8, or Δ9. Cells were then treated with HK media for 24 h and viability was quantified (*p<0.05, **p<0.01 and ***p<0.001 as compared to mut-Htt/Ctrl co-transfected cells; ###*p*<0.001 as compared to GFP; ^*p*<0.05 as compared to mut-HTT/SIRT1 co-transfected cells; n = 3).

In order to narrow the region within SIRT1 conferring neuroprotection, we expressed ten different deletion mutants, termed Δ1-Δ10, that each contain ~50–70 sequential amino acid deletions spanning the SIRT1 protein (Fig 1A), into CGNs. When kept in media supplemented with high extra cellular potassium (HK), CGNs remain healthy and viable. However, when switched to media containing low levels of potassium (LK), roughly 50% of the neurons undergo apoptosis within 24 h. As shown in Fig 1B, Δ4-Δ7, which span the central catalytic domain, were just as protective against LK-induced apoptosis as full length SIRT1. However, it has been reported that by itself the catalytic domain has little-to-no activity and is instead dependent on its flanking terminals [36–39]. We find that deletion of any part of SIRT1’s N-terminal (Δ1-Δ3), which contain two motifs that function by regulating the catalytic rate [36, 37], still had no influence on its protective effect (Fig 1B). Within SIRT1’s C-terminal, a 25 amino acid region (631–655), known as the ESA (*E*ssential for *S*IRT1 *A*ctivity), has been identified that functions as a switch that binds SIRT1’s catalytic domain and turns on its deacetylase activity [39]. Deletion of the ESA motif eliminates the catalytic activity of SIRT1 [39]. Ectopic expression of the Δ9 construct, which lacks this ESA region, is just as protective as full length SIRT1 (Fig 1B). We find, however, that expression of the Δ8 construct, a largely uncharacterized 67 amino acid region just C-terminal to the catalytic domain, exhibited a significantly reduced protective effect by ~22.5 ± 5.5% compared to full length SIRT1 (Fig 1B). In conjunction with our previous data obtained using pharmacological inhibitors and catalytically-dead forms of SIRT1 [5], these results provided additional support to suggest that SIRT1 protection against LK-induced apoptosis in CGNs is deacetylase-independent.

SIRT1 is protective in both *in vitro* and *in vivo* paradigms of Huntington’s disease [9, 41]. We have previously reported that otherwise healthy CGNs kept alive by HK treatment are susceptible to mut-Htt toxicity [42–45]. To determine if SIRT1 can additionally protect against mut-Htt in a deacetylase-independent manner, we co-expressed mut-HTT with various SIRT1 deletion constructs into CGNs and treated them with HK media. When expressed either alone or with a control vector (Ctrl), mut-Htt induced death in these neurons (Fig 1C). As expected, full length SIRT1 was able to rescue mut-Htt toxicity (Fig 1C). Similarly, protection was observed by co-expression with Δ1, Δ4, Δ6, Δ7, and Δ9, as well as a deacetylase-deficient mutant, H363Y, that is located within the Δ5 region. Δ8, however, was unable to protect, thus suggesting that SIRT1 can block mut-Htt toxicity *in vitro* without its catalytic activity (Fig 1C).

### Neuroprotection by SIRT1 is blocked by the pharmacological inhibition of HDACs

**Fig 2:**
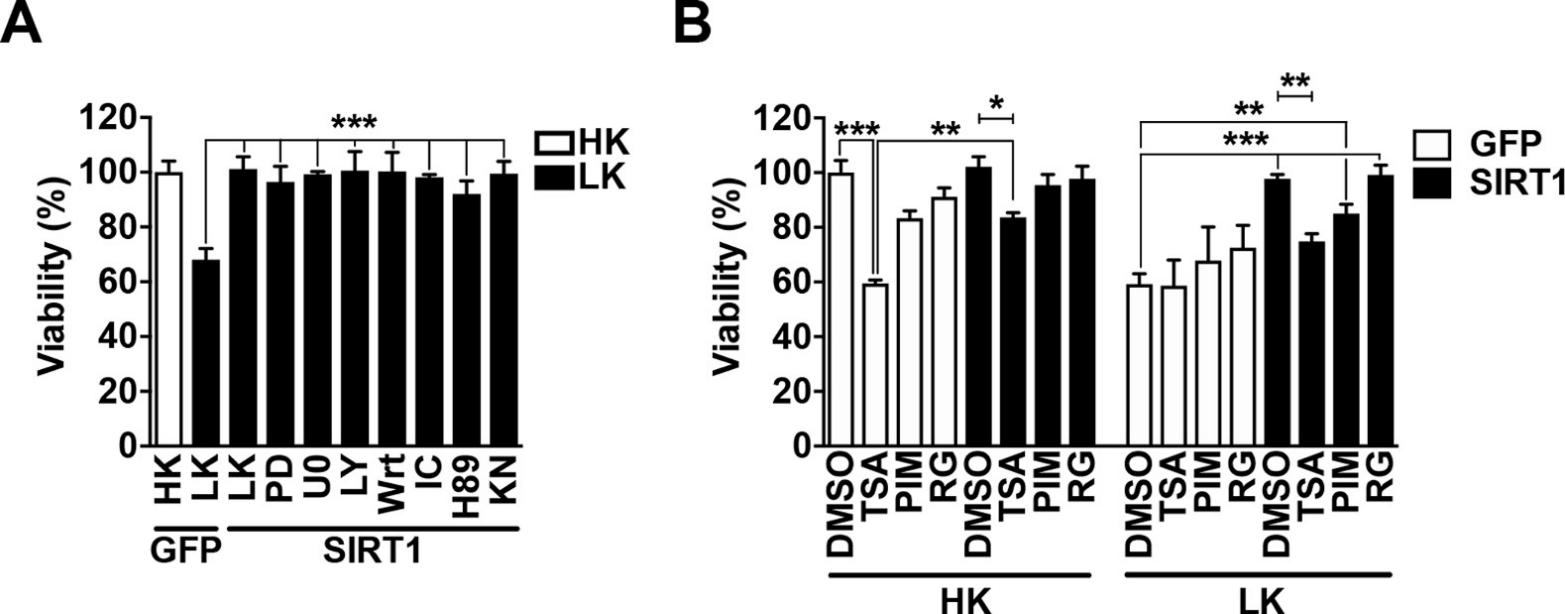
SIRT1 protection is blocked by inhibition of HDACs. **A.** GFP or SIRT1-Flag were ectopically expressed in CGNs for 24 h. Media was then switch to that containing either HK, LK, or LK supplemented with the following inhibitors: PD98059 (MEK inhibitor), U0126 (MEK inhibitor), LY294002 (PI3-K inhibitor), Wortmannin (PI3-K inhibitor), IC261 (CK1 inhibitor), H89 (PKA inhibitor), KN-62 (CaMKII inhibitor) (***p<0.001; n = 3). **B.** CGNs were transfected and treated as described in (A) as indicated with the following inhibitors: trichostatin A (TSA), pimelic diphenylamide 106 (PIM), or RGFP966 (*p<0.05, **p<0.01, ***p<0.001; n = 3).

As SIRT1’s neuroprotection is dependent on the Δ8 region (residues 542–609), we sought to determine how this stretch is providing this effect. SIRT1 activity can be regulated post-translationally [46]. We hypothesized that SIRT1 is post-translationally modified through phosphorylation by a pro-survival signaling pathway. To answer this, SIRT1 was expressed into CGNs and treated with either LK media or LK media supplemented with pharmacological inhibitors targeting well-known pro-survival molecules. Inhibition of the MEK/ERK or PI3-K pathways as well as casein kinase 1, protein kinase A, or Ca2+/calmodulin kinase II, did not hinder SIRT1 protection (Fig 2A). In separate experiments we verified that the pharmacological inhibitors were effective at the concentration we used (data not shown).

HDACs are well known to form and work in protein complexes, often with one another [1]. Since sirtuin inhibitors, and more specifically the broad-spectrum nicotinamide that targets the deacetylase activity of all sirtuin family members, did not affect SIRT1 protection [5], we investigated if SIRT1 was dependent on a classical HDAC. Interestingly, we found that when CGNs under LK conditions were treated with trichostatin A (TSA), a well-known broad-spectrum classical HDAC inhibitor, SIRT1-mediated protection was significantly decreased by ~22 ± 2.78% (Fig 2B). TSA treatment is highly toxic to CGNs kept alive by HK media, and under these conditions, SIRT1’s protective ability was also significantly decreased by ~18 ± 1.74% (Fig 2B).

Due to TSA’s broad-spectrum nature, we utilized additional inhibitors to determine if SIRT1 was dependent on a specific HDAC. As Class IIa HDACs (HDACs 4, 5, 7, and 9) have been shown to possess little catalytic activity on their own [47], we treated SIRT1 expressing CGNs with a Class I specific inhibitor, pimelic diphenylamide 106 (PIM) [31]. We found that while SIRT1 still retained a protective effect against LK, it was reduced by ~13 ± 3.36% (Fig 2B). Since PIM targets HDACs 1, 2 and 3, with a preference for HDAC3, and little specificity for HDAC8 [31], we further treated CGNs with a widely used and highly selective HDAC3 inhibitor, RGFP966 [48–50]. However, we find that the inhibition of HDAC3 activity did not block SIRT1-mediated protection (Fig 2B). Together, these pharmacological results suggest that SIRT1 neuroprotection is dependent on a Class I HDAC.

### SIRT1 interacts with HDAC1

**Fig 3:**
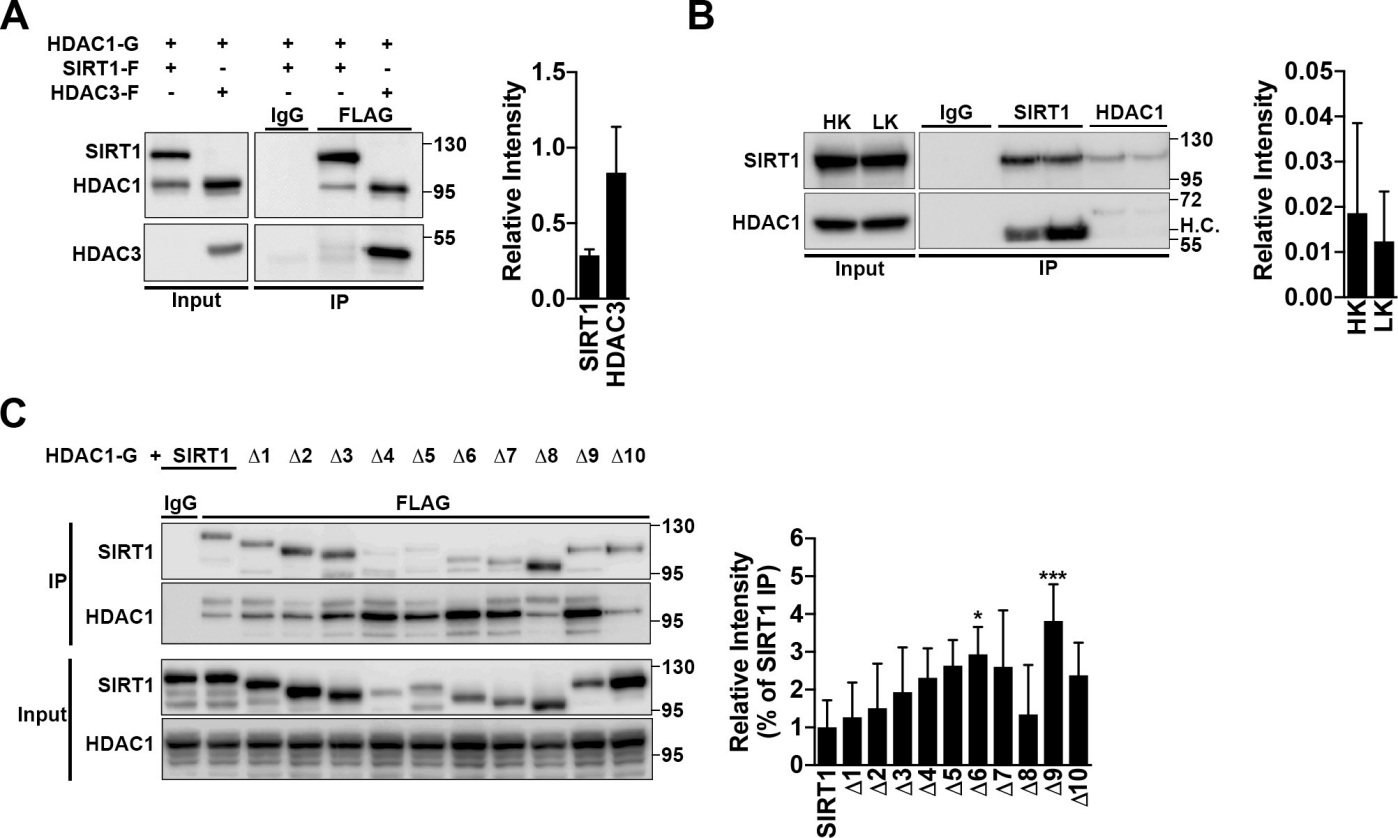
SIRT1 interacts with HDAC1. **A**. Confirmation that SIRT1 and HDAC1 interact. HDAC1-GFP was co-expressed in a 1:1 ratio with either SIRT1-Flag or HDAC3-Flag into HEK293T cells. 48 h later, cells were lysed and immunoprecipitation (IP) was performed as indicated with either an IgG or Flag antibody. Membranes were probed with a GFP antibody to detect HDAC1 pulldown and then reprobed with a Flag antibody to check for proper pulldown of SIRT1 and HDAC3. Input represents 10% of whole cell lysate used for IP. Graph represents densitometry of HDAC1 pulldown from four independent experiments. **B.** Endogenous immunoprecipitation was performed on CGNs treated with HK/LK media for 6 h, with either an IgG, SIRT1, or HDAC1 antibody. H.C.: heavy chain. Graph represents densitometry from four independent experiments. **C**. Immunoprecipitation was performed as described in (A) with HDAC1-GFP co-expressed with flag-tagged SIRT1 or Δ1-Δ10. Pulldown was performed with either an IgG or Flag antibody. Membranes were initially probed with a GFP antibody and then stripped and reprobed for Flag. Graph represents densitometry (one-way ANOVA with Dunnett’s multiple comparisons posttest; *p<0.05, ***p<0.001) from five independent experiments and normalized to pulldown of full length SIRT1.

We have previously reported that HDAC3 is highly and selectively toxic to neurons [34], and have further shown that HDAC1 can be either protective or toxic, depending on its ability or not to interact and cooperate with HDAC3 [35]. As SIRT1 protection is hindered by inhibitors that target HDACs 1 and 3, and since inhibition of HDAC3 protects neurons [34] and does not inhibit SIRT1, we turned our attention to HDAC1. We first confirmed that the two interacted through co-immunoprecipitation, and although the interaction was weaker than that of HDAC1 and HDAC3, SIRT1 did indeed pull down HDAC1 (Fig 3A). We next examined if this interaction occurred endogenously in CGNs. As shown in Fig 3B, following a 6 h HK/LK treatment, a time at which CGNs have committed to undergo apoptosis under LK conditions, we find that HDAC1 was able to pull down SIRT1 under both HK and LK. However there was no significant difference between the conditions (Fig 3B). While SIRT1 has been shown to physically interact with the N-terminal of HDAC1 [51], we wanted to narrow the interacting region within SIRT1, which we hypothesized to be Δ8. Interestingly, HDAC1 interacted with Δ8, as well as Δ1-Δ3 and Δ10, to a similar extent that it does with full length SIRT1 (Fig 3C). Furthermore, we find that HDAC1 interacted greater with deletions constructs that span regions important for SIRT1’s deacetylase activity (Δ4-Δ7 and Δ9), than it did with full length SIRT1 (Fig 3B). These findings, in conjunction with the previous inhibitor results, suggest that association with HDAC1 contributes to the catalytic-independent protection effect of SIRT1 and that association is enhanced by SIRT1 catalytic activity. However, the finding that Δ8 associates with HDAC1 to the same extent as the protective SIRT1 constructs suggests that association with HDAC1 may not be required for neuroprotection by SIRT1. Alternatively, it is possible that factors other than association with HDAC1 are also necessary for SIRT1-mediated neuroprotection.

### SIRT1 requires HDAC1 for neuroprotection

**Fig 4:**
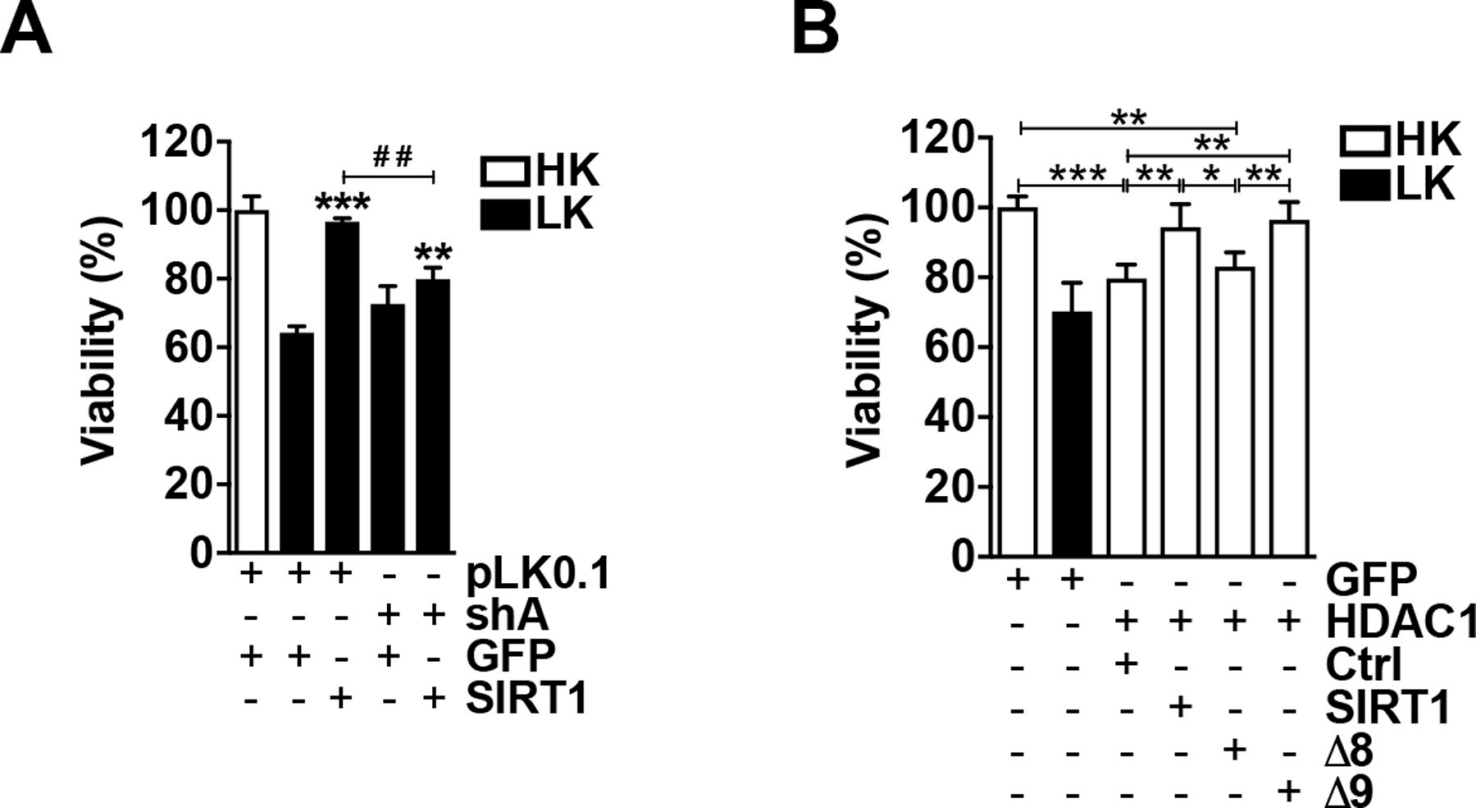
SIRT1 requires HDAC1 to protect CGNs against apoptosis. **A**. SIRT1 requires HDAC1. GFP or SIRT1-Flag were co-expressed in a 1:2 ratio with either a control shRNA (pLK0.1) or a distinct HDAC1 shRNA (sh A) (**p<0.01 and ***p<0.001 as compared to GFP/pLK0.1 treated with LK; ##*p*<0.01 as compared to SIRT1/pLK0.1 treated with LK; n = 3). **B**. SIRT1 and Δ9, but not Δ8, rescue HDAC1 toxicity. CGNs were transfected with GFP or co-transfected with HDAC1-GFP and either a control vector (Ctrl, 3xFLAG), SIRT1-Flag, Δ8-Flag, or Δ9-Flag in a 1:2 ratio (*p<0.05, **p<0.01, ***p<0.001; n = 3).

Since SIRT1-mediated protection is negatively affected by classical HDAC inhibition, and deletion constructs targeting regions important its deacetylase activity interacted greater with HDAC1, we reasoned that SIRT1 needed HDAC1 for its protective effect. We investigated this by knocking down the endogenous levels of HDAC1 with an shRNA we have previously shown as effective against HDAC1 in neurons [35]. As shown in Fig 4A, while SIRT1 still protected against LK-induced cell death in cells depleted of HDAC1, this was significantly reduced by ~17 ± 3.4%, suggesting that it required the presence of HDAC1 for its protective effect. Ectopic expression of HDAC1 alone is toxic to neurons since it can interact with and utilize HDAC3 deacetylase activity [35]. Because HDAC1 interacted with full length SIRT1 and Δ9 and since SIRT1 might depend on HDAC1, we further reasoned that they would be able to rescue HDAC1-induced toxicity when expressed together. Indeed, we found that when co-expressed with either full length SIRT1 or Δ9, HDAC1 was no longer toxic to CGNs (Fig 4A). Since Δ8 interacted with HDAC1 to a similar degree as full length SIRT1 but had a reduced ability to protect against LK-induced apoptosis, we tested whether it could block HDAC1 toxicity. However, when expressed together, HDAC1 still induced death in otherwise healthy neurons (Fig 4A). This suggested that either SIRT1 could block HDAC1-induced death or that the two might cooperate to protect against neuronal death. Taken together, these results indicate that although its association is not sufficient, HDAC1 is required for SIRT1-mediated neuroprotection.

### HDAC1 activity is not needed for SIRT1’s interaction or protective effect

**Fig 5:**
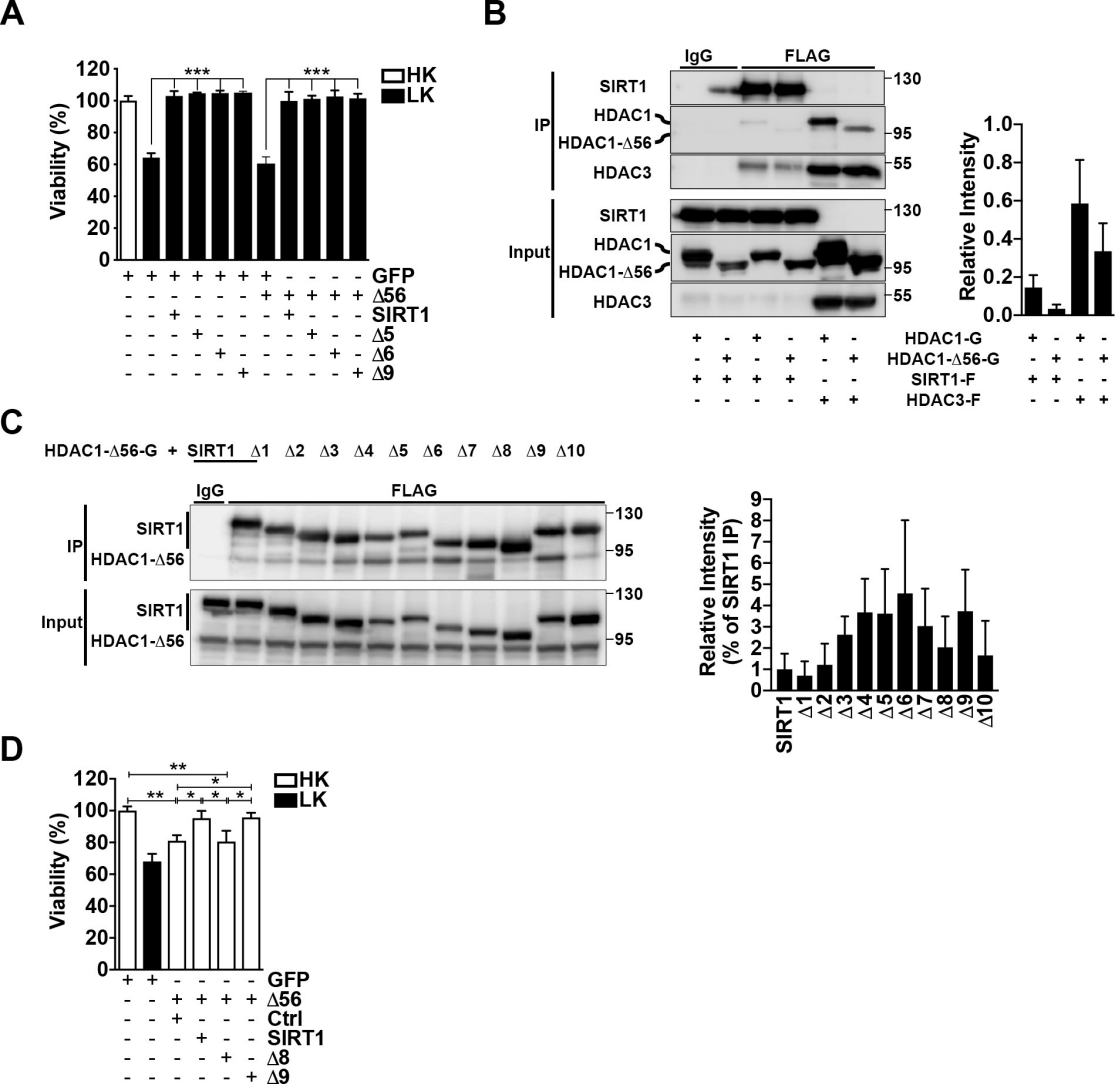
SIRT1 does not require HDAC1 activity. **A**. CGNs were expressed with GFP alone or co-expressed with SIRT1, Δ5, Δ6, or Δ9 in a 1:2 ratio with GFP or Δ56 and then treated as indicated (***p<0.001; n = 3). **B.** SIRT1-Flag or HDAC3-Flag were each expressed in a 1:1 ratio with either HDAC1-GFP or Δ56-GFP into HEK293T cells for 48 h after which immunoprecipitation was performed with either and IgG or Flag antibody. Western blots were initially probed for GFP and subsequently probed for Flag. Graph shows densitometry from four independent experiments. **C**. Δ56 was expressed into HEK293T cells in a 1:1 ratio with SIRT1 or Δ1-Δ10 for 48 h. Immunoprecipitation was performed as described in (B). Graph shows densitometry (one-way ANOVA with Dunnett’s multiple comparisons posttest) from five independent experiments. CGNs were co-transfected with GFP or Δ56-GFP and either a control vector (Ctrl, 3xFLAG), SIRT1-Flag, Δ8-Flag, or Δ9-Flag in a 1:2 ratio (*p<0.05, **p<0.01; n = 3).

The combination of our inhibitor data, interaction studies, and SIRT1’s reduced protective effect when endogenous HDAC1 is depleted from CGNs indicated that SIRT1 deacetylase-independent protection is acquired through HDAC1 activity, which we subsequently investigated. We have previously characterized an HDAC1 deletion mutant lacking its first 56 amino acids, termed Δ56, that possesses no catalytic activity [35]. Interestingly, when SIRT1 was co-expressed with this HDAC1 mutant, it still retained its protective ability, indicating that HDAC1 activity is not necessary for SIRT1 protection (Fig 5A). We further found that Δ5, Δ6, and Δ9, each of which interacted with HDAC1 to a greater degree than full length SIRT1, were additionally still protective in the presence of Δ56 (Fig 5A). We proceeded to determine if SIRT1 still retained the ability to interact with Δ56. Following co-expression into HEK293T cells, SIRT1 pulled down Δ56, albeit to a lesser degree than full length HDAC1 (Fig 5B). Further co-immunoprecipitation analysis revealed that Δ56 also interacted with the full panel of SIRT1 deletion constructs in a similar manner to that of full length HDAC1 (Fig 5C). HDAC1-Δ56, just like full length HDAC1, is toxic to healthy CGNs when expressed alone because it can interact with and utilize HDAC3’s deacetylase activity [35]. We hypothesized that due to their ability to interact, SIRT1 and Δ9 could further block Δ56-induced death. As was observed with full length HDAC1, SIRT1 and Δ9 both protected against apoptosis due to Δ56 expression (Fig 5D). These results indicate that while HDAC1 is needed for SIRT1’s protective ability, its deacetylase activity is not. We extended our investigation to determine if Δ8 had the ability to block Δ56 toxicity, however as shown in Fig 5D it failed to do so. This result raises the possibility that rather than a loss of SIRT1-mediated neuroprotection, the deletion of the Δ8 region may confer on SIRT1 a toxic gain-of-function effect.

### Loss of the Δ8 region is toxic to neurons

**Fig 6:**
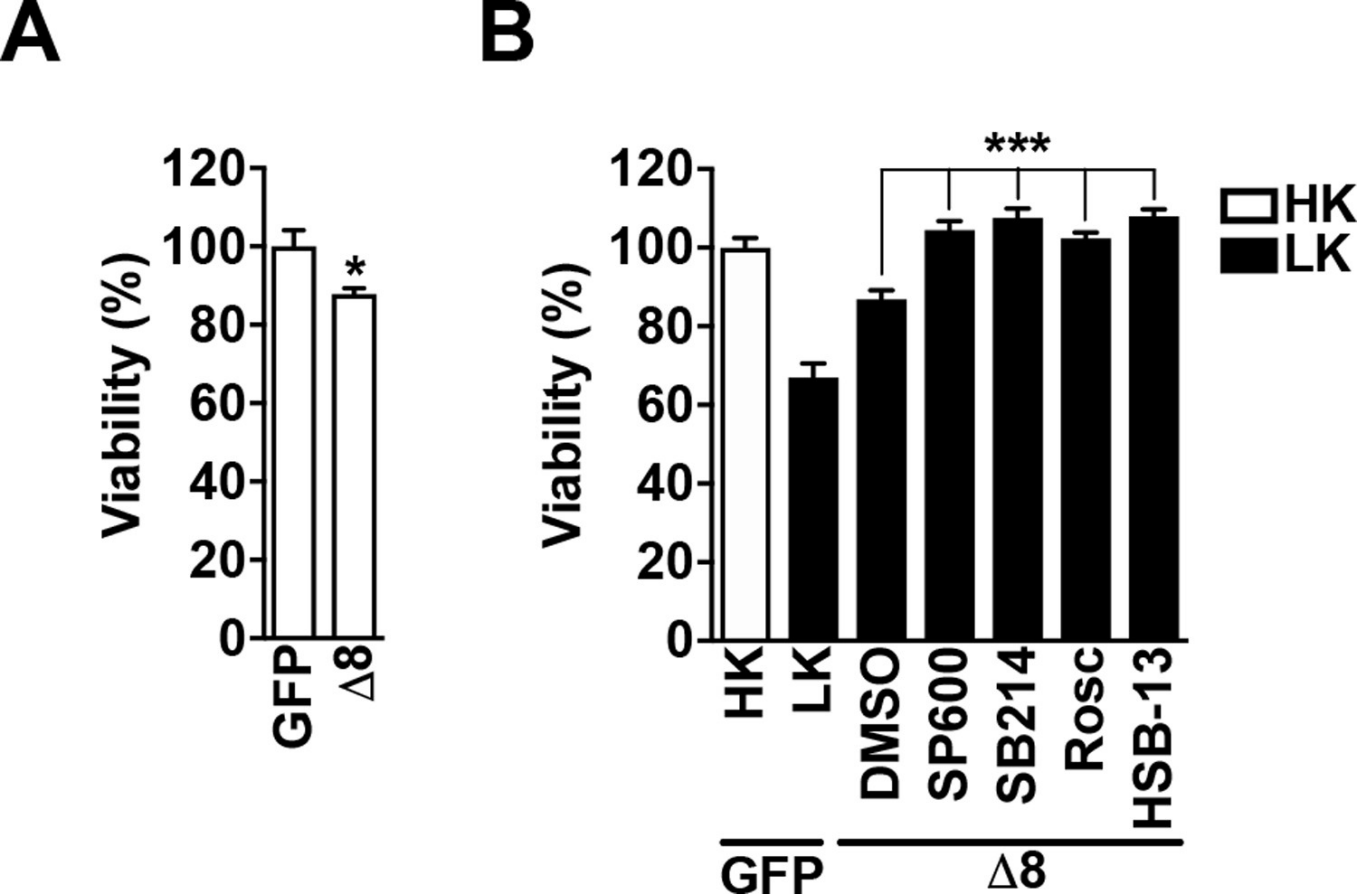
Loss of the Δ8 region is toxic to neurons. **A.** Expression of Δ8 induced death in otherwise healthy neurons. GFP or Δ8 were expressed into CGNs and then treated with HK media for 24 h (*p<0.05; n = 3). **B.** CGNs were transfected with GFP or Δ8. Neurons were then treated with either HK, LK, or LK media supplemented with the following for 24 h: DMSO, SP600125 (JNK inhibitor), Roscovitine (CDK inhibitor), SB216763 (GSK3 inhibitor), HSB-13 (***p<0.001; n = 3).

To determine if the loss of the Δ8 region instilled a toxic gain-of-function effect, we examined if this construct had an effect on otherwise healthy neurons kept alive under HK-treated conditions. Interestingly, we found that it significantly induced death by ~13 ± 1.5% when compared to a GFP control (7A). To understand how the Δ8 construct promotes neuronal death, it was expressed into CGNs and subsequently treated with inhibitors targeting signaling pathways known to be pro-apoptotic in neurons. We find that Δ8’s decreased protective ability is rescued by inhibition of c-Jun N-terminal kinase (JNK), glycogen synthase kinase 3 (GSK3), and cyclin dependent kinases (CDKs) (Fig 6B). We have previously reported that a compound referred to as HSB-13 is highly protective to neurons [29]. While it does not target JNK, HSB-13 can protect CGNs against apoptosis through the inhibition of GSK3α/β and CDKs, namely CDK1/cyclin A1 and CDK2/cyclin A1[29]. Following treatment with HSB-13, Δ8’s toxicity is completely abolished (Fig 6B). These findings suggest that the neurotoxic effect of SIRT1-Δ8 involves activation of JNK, GSK3β and CDKs.

## Discussion

It is generally believed that the many cellular functions of SIRT1 are mediated through its NAD+-dependent catalytic activity. But whether SIRT1 can function independent of its catalytic activity has not been rigorously examined. One well-described activity of SIRT1 is its ability to protect neurons both in cell culture and animal models of neurodegeneration, particularly when overexpressed [7, 9–11, 19]. We previously described that two separate point-mutant forms of SIRT1 lacking in catalytic activity, H355A and H363Y, were just as protective as wild-type SIRT1 in CGNs induced to undergo apoptosis by withdrawal of depolarizing stimuli [5]. Additionally, neither of two structurally distinct pharmacological SIRT1 inhibitors reduced the ability of ectopically expressed SIRT1 to protect neurons [5]. To rigorously confirm that SIRT1 could indeed protect independently of its catalytic activity, we have now analyzed additional mutants lacking relatively large portions of the catalytic domain as well as regions outside of it. Consistent with our previous data using point-mutants, the SIRT1 deletion mutants lacking portions of the catalytic domains were also neuroprotective (Fig 1B). The most important region for SIRT1’s deacetylase activity is the ESA motif mapped to residues 631–655 within SIRT1’s C-terminal, which interacts with and functions as an "on switch" for the deacetylase core [39]. We find that expression of the Δ9 construct, which lacks the ESA, still protects neurons (Fig 1B). In addition to the C-terminal, other studies have described that the N-terminal of SIRT1 is necessary for its catalytic activity [36–39]. Within the N-terminal lie two motifs, Motif A (residues 1–52) and Motif B (residues 163–221). These motifs operate independently of one another and may function through association with each other in trans leading to SIRT1 dimerization, or in cis to enhance substrate binding and SIRT1 catalytic activity as a monomer [36]. Motif B also contains a region that is targeted by small molecule activators of SIRT1 catalytic activity [52, 53]. We previously reported that a SIRT1 construct lacking its first 81 amino acids, and thus Motif A, was just as protective as the full-length protein [5]. Here we show that deletion of either Motif A or B with constructs Δ1 and Δ3, respectively, did not interfere with protection (Fig 1B). Collectively, along with our previous study [5], our results now conclusively demonstrate that SIRT1 can protect neurons against apoptosis through a mechanism that is independent of its catalytic activity. A similar conclusion was reached by Singh et al. who described that a catalytically-dead point-mutant form, of SIRT1, H363Y, was protective in a cell culture model of Parkinson’s disease [11]. Other studies using other non-neuronal cell types have described that some other properties and functions of SIRT1 are also independent of its catalytic activity [23–25].

As observed in LK-induced death, we found that SIRT1 deletion-mutants Δ4–Δ7 and Δ9, as well as the point mutant H363Y protected CGNs against mut-Htt neurotoxicity suggesting that the catalytic activity-independent mechanism of protection by SIRT1 is also effective in models of proteotoxic neurodegeneration (Fig 1C). Our results appear to contradict those of Jeong et al. who reported that SIRT1-H363Y was incapable of protecting neurons against mut-Htt toxicity [21]. The reason for the difference is unclear. It may be noted that lentivirus was used for expression in the Jeong study whereas we used plasmid expression. Moreover, our results come from CGNs whereas the Jeong study used cortical neurons. In our experiments, however, rat cultures were used whereas Jeong et al. used cortical neurons cultured from mice. It is possible that one or more of these culture conditions could be responsible for the difference in results. While elevating SIRT1 has been shown to improve behavioral deficits in N171-82Q and R6/2 mice, it has not been established that these *in vivo* effects require the catalytic activity of SIRT1 [9, 21].

SIRT1 neuroprotection in CGNs is HDAC1-dependent as it is reduced or blocked by pharmacological inhibition of Class I HDACs, but not HDAC3 (Fig 2B), and shRNA-mediated depletion of endogenous HDAC1 (Fig 4A). Furthermore, SIRT1 mutants containing deletions of regions necessary for catalytic activity interact with HDAC1 to a much greater degree than full length SIRT1 (Fig 3C). These results would suggest that SIRT1 protects neurons through acquiring and utilizing HDAC1 activity, however, the presence of the deacetylase dead HDAC1-Δ56 does not hinder this protection (Fig 5A). Moreover, SIRT1 and its mutants can interact with and protect against HDAC1-Δ56 in a similar manner seen with full length SIRT1 (Fig 5B–5D). Further support for the lack of HDAC1 activity needed is that N-terminal affinity tags, of which both HDAC1 and HDAC1-Δ56 possess, have been shown to interfere with HDAC1 catalytic activity [51]. Together, our results suggest that SIRT1 and HDAC1 work together to protect CGNs through a deacetylase-independent manner.

Functional cooperation between SIRT1 and HDAC1 has been reported following double-stranded DNA strand-breaks [51]. A few other studies performed in non-neuronal systems have also shown cooperation between SIRT1 and HDAC1 [51, 54, 55]. Although we and other labs have described that HDAC1 has neuroprotective activity, it is also known that HDAC1 can promote neuronal death [35, 51, 56, 57]. We previously described that HDAC3 is a neurotoxic protein and that its neurotoxicity involves interaction with HDAC1 [35]. In contrast to HDAC1, which is normally exclusively nuclear, HDAC3 localizes to both the cytoplasm and the nucleus. It is tempting to speculate that translocation of HDAC1 to the cytoplasm, due to reduced interaction with SIRT1 that is also nuclear, permits it to interact with HDAC3 thus promoting neuronal death. Indeed, previous studies have described that cytoplasmic translocation of HDAC1 promotes degenerative effects in neurons [57, 58]. Also possible is that an HDAC1 and HDAC3 interaction occurs in the nucleus but is promoted by post-translational modifications. We have previously described that the neurotoxic effect of HDAC3 requires its phosphorylation by GSK3β[34, 35]. A promising possibility is that SIRT1 sequesters HDAC1 from HDAC3 and utilizes it for neuroprotection. Although our study indicates that SIRT1 and HDAC1 might cooperate together in a deacetylase-independent manner in CGNs, it remains unclear if the two function as part of a larger complex. Dobbin et al have shown that SIRT1 and HDAC1 interact through HDAC1’s N-terminal [51], however we find that SIRT1 can pull down an HDAC1 lacking its first 56 amino acids. Thus it is possible that the two complex together with other protein(s). Alternatively, it is possible that there are two separate binding sites for HDAC1 within SIRT1. Supporting this latter possibility is our finding that all the SIRT1 deletion constructs interact with HDAC1.

An intriguing aspect of our study is the observation that the neuroprotective effect of SIRT1 is lower in the presence of HDAC inhibitors. Although the most straightforward explanation for this is that SIRT1 requires the catalytic activity of a Class I HDAC like HDAC1, our investigations reveals that this may not be the case. Indeed, neither association of SIRT1 with HDAC1 nor its neuroprotective effect is affected when a catalytically-dead form of HDAC1 is utilized. This raises the possibility that another Class I HDAC could be responsible. One candidate HDAC is HDAC3. Previous work from our lab has found that HDAC3 plays a critical role in the development and functioning of the brain [59] and that it can be converted into a neurotoxic protein upon its phosphorylation by GSK3β and interaction with HDAC1 [35]. However, a widely used and highly selective inhibitor of HDAC3, RGFP966, has no effect on neuroprotection by SIRT1, arguing against the need for HDAC3 in the protective action of SIRT1. While it is possible that SIRT1 may cooperate with other Class I HDACs like HDAC2 and HDAC8, it is more likely that Class I HDAC inhibitors have a toxic effect that cannot be prevented by SIRT1 overexpression. We and others have previously described toxicity by HDAC inhibitors, including TSA [33, 60, 61].

Deletion of the region spanning residues 542–609 (Δ8) in SIRT1 significantly reduced its neuroprotective ability against LK-induced apoptosis and was mildly toxic to otherwise healthy neurons under HK conditions (Figs 1B and 6A). Furthermore, while the Δ8 construct interacts with both HDAC1 and HDAC1-Δ56, it is unable to rescue their expression-induced toxicity in HK. We explored the possibility that this region contains residues that are post-translationally modified through phosphorylation by kinases associated with neuroprotection. However, inhibition of three separate kinases, CK1, PKA and CaMKII, or the MEK-ERK and PI3-K signaling pathways, did not affect SIRT1-mediated neuroprotection (Fig 2A). We found, however, that GSK3 inhibition blocked the reduction in protection by Δ8 (Fig 6B). Both HDAC1 and HDAC3 require active GSK3β for their toxic function in CGNs [34, 35]. It is plausible that GSK3 inhibition frees HDAC1 from its toxic HDAC3 interaction thereby allowing a protective relationship with SIRT1. Additionally, we further show that CDK inhibition also blocked Δ8’s reduced protective ability. In conjunction with the mild toxicity to HK, this suggests that rather than losing neuroprotective activity, Δ8 may gain a “toxic” cell cycle stimulating activity. It is well established that abortive re-entry of neurons into the cell cycle contributes to neurodegeneration both *in vitro* and *in vivo* [44, 62, 63]. We have also recently shown that a SIRT1-interacting protein, JAZ, protects neurons through the induction of p21 expression and cell cycle suppression [44]. Thus more work is required to fully understand the mechanism by which the loss of the Δ8 region leads to SIRT1 losing its protective function.

In conclusion, our studies show that elevating SIRT1 expression is neuroprotective in both proteotoxic and non-proteotoxic models of neuronal death and that this does not require its catalytic activity. We propose that SIRT1 interacts and cooperates with HDAC1 in a further deacetylase-independent manner. While SIRT1-mediated neuroprotection is compromised by the loss of a C-terminal region, more work is required to determine whether this is due to a loss of protection or a gain of a toxic effect and to fully delineate the mechanisms involved. Based on neuroprotection by SIRT1 in cell culture and *in vivo* models of neurodegenerative disease it has been suggested that elevating SIRT1 activity could have benefits in neurodegenerative diseases. Promoting a SIRT1-HDAC1 interaction could represent another therapeutic approach for these diseases.
